# Functional Aesthetic Occlusal Plane (FAOP)

**DOI:** 10.1590/2177-6709.21.4.114-125.sar

**Published:** 2016

**Authors:** Carlos Alexandre Câmara, Renato Parsekian Martins

**Affiliations:** 1 Private Practice, Natal, Rio Grande do Norte and Recife, Pernambuco, Brazil.; 2 Adjunct professor, Universidade Estadual Paulista (UNESP), School of Dentistry, Orthodontics Program, Araraquara, São Paulo, Brazil.

**Keywords:** Aesthetics, Smile, Dental occlusion, Orthodontics.

## Abstract

**Introduction:**

A reasonable exposure of incisors and gingival tissues is generally considered more attractive than excess or lack of exposure. A reasonable gingival exposure is considered to be around 0 to 2 mm when smiling and 2-4 mm exposure of the maxillary incisor edge when the lips are at rest.

**Objective:**

The aim of this paper is to present the Functional Aesthetic Occlusal Plane (FAOP), which aims to help in the diagnosis of the relationships established among molars, incisors and the upper lip.

**Conclusion:**

FAOP can complement an existing and established orthodontic treatment plan, facilitating the visualization of functional and aesthetic demands by giving a greater focus on the position of incisors in the relationship established among the incisors, molars and the upper lip stomion.

## RESUMO

**Introdução:**

uma exposição razoável dos incisivos e da gengiva é, geralmente, considerada mais atraente do que quando há excesso ou falta de exposição, tanto de dentes quanto de gengiva. Essa faixa razoável encontra-se em torno de 0 a 2mm de exposição gengival durante o sorriso e de 2 a 4mm de exposição da borda incisal dos incisivos superiores em repouso.

**Objetivo:**

o objetivo do presente trabalho é apresentar o plano oclusal estético funcional (POEF), que tem por propósito auxiliar no diagnóstico da relação de posicionamento entre molares, incisivos e ponto estômio do lábio superior.

**Conclusão:**

o POEF pode ser um complemento ao plano de tratamento ortodôntico já existente e estabelecido, facilitando a visualização das demandas estéticas e funcionais, dando um enfoque maior à posição dos incisivos na relação entre os incisivos, molares e o ponto estômio do lábio superior.

## INTRODUCTION

Incisors exposure during speech, at smiling and when the lips are at rest is an important factor in facial aesthetics, as it influences perception of the human face. A reasonable exposure of incisors and gingiva is generally considered more attractive than when there is excess or lack of exposure of teeth and/of gingiva.^1^ The literature establishes that this reasonable exposure is considered to be around a 0-to-2 mm exposure of the gingiva at smiling^2^ ^,^ ^3^ and a 2-to-4 mm exposure of the edge of maxillary incisors when at rest^4^. One important morphological characteristic is that the relative position of incisors changes with time. Maxillary incisor exposure decreases with age, while mandibular incisor exposure increases.^5^ ^,^ ^6^ In other words, younger individuals have greater maxillary incisor exposure, while older people have greater mandibular incisor exposure. This information is important because the amount of exposure of maxillary and mandibular incisors gives us an idea of the age of individuals and points out the aesthetic needs of patients according to age. 

Fortunately, today, there are various means to modify the position of maxillary incisors in a reliable manner, either by extruding^7^ or intruding them.^8^ The same applies to mandibular incisors which can also be moved with predictability.^9^ On the other hand, posterior teeth, especially molars, do not seem to substantially change their vertical position over time. Albeit, there are changes in the vertical position of molars during growth; however, this effect seems to be markedly higher in the pre-pubertal and pubertal stages than in adulthood.^10^ Rapid and pronounced wear on tooth structures can change the vertical relationship of molars in individuals without growth, but one should consider that this is unusual and should be seen as abnormal. Even though the advent of temporary skeletal anchorage devices have made extensive movement of posterior teeth possible, there is no strong evidence that large vertical orthodontic movements in adults remain over time. There have been relapses of orthodontic treatment in adults with vertical movements reported in the literature,^11^ ^,^ ^12^ which tends to support the idea that this relationship (vertical) of posterior teeth appears to be stable if they are kept in their original position in individuals without growth, and without changing the free space between them.

Understanding the dynamics of vertical positioning of incisors and molars leads to the conclusion that an occlusal plane that includes these dental structures also undergoes change over time. Thus, one might ask: why not add an aesthetic component to the functional occlusal plane which also takes into account the positioning of teeth over time? Which leads to a second question: which aesthetic component would that be?

Since the exposure of incisors in relation to the lips is an important aesthetic factor, the upper lip stomion should be the component chosen to be added to this Functional Aesthetic Occlusal Plane (FAOP). Thus, the use of an occlusal plane that takes into account aesthetic and functional factors can fill an important gap in orthodontic diagnosis and treatment planning, which is to create a bridge between aesthetic and occlusal factors. However, for this function/aesthetic connection to be useful, it is necessary to establish a correlation with the face. Thus, the FAOP should be used in conjunction with the facial profile, which is the interactive relationship between teeth and face.

Therefore, this article aims to describe the Functional Aesthetic Occlusal Plane (FAOP), which is a diagnostic reference that takes into account the vertical dimension established by the occlusion of molars and the vertical positioning (aesthetic) of incisors in relation to the upper lip stomion, and uses facial proportions as reference indicators. 

### Functional Aesthetic Occlusal Plane

**Figure 1:**
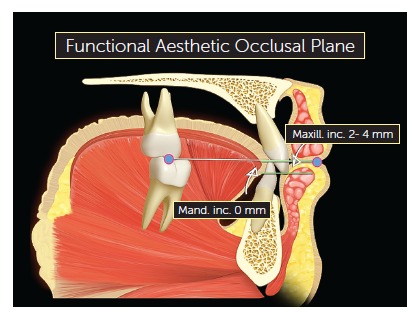
Functional aesthetic occlusal plane. The incisal edge of maxillary incisors should be 2-4 mm below the FAOP and the incisal edge of mandibular incisors should touch the plane.

**Figure 2:**
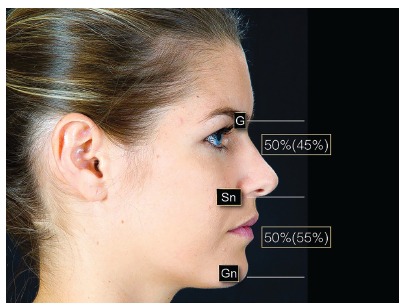
Proportions between the middle thirds (Glabella-Subnasale) and lower middle third. (Subnasale-Gnathion) 50%(45%) / 50%(55%).

The average point of contact between maxillary and mandibular first molars and the upper lip stomion is taken as a reference. Then a line is drawn between these two points to determine the FAOP (Fig 1). The edge of the maxillary incisor (or planned, if it is to be restored) should be positioned 2-4 mm below this plane; while the edge of mandibular incisors should touch this plane. These measures serve as a reference and aim to facilitate understanding of the functional aesthetic relation of anterior teeth; however, they will be subject to slight variations depending on age, dental proportions, overbite and the smile line. The facial proportions used in FAOP between the distances of Glabella (G)-Subnasale (Sn) to Sn-Gnathion (Gn) are 50-45% to 50-55%, as a basis for the evaluation of good facial proportions^13^ (Fig 2). The FAOP takes into consideration the aesthetic positioning of maxillary and mandibular incisors in relation to the lips without changing the vertical position of molars, without affecting any functions and with connection to the facial profile. Here the use of FAOP is demonstrated in two cases, one for open bite and the other for overbite. 

### Functional Aesthetic Occlusion Plane for open bite treatment

Female 16-year-old patient had Class III malocclusion with an anterior open bite of -2 mm between incisors. Although functional issues, such as difficulty biting in the anterior region, were involved, her main complaint was aesthetical; she did not like her smile and argued that it was aged.

**Figure 3:**
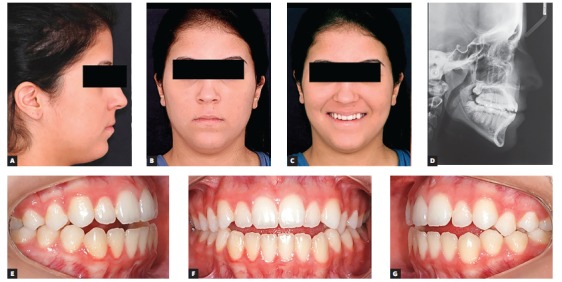
Initial photographs: extraoral (A, B, C), radiograph (D) and intraoral (E, F, G).

**Figure 4:**
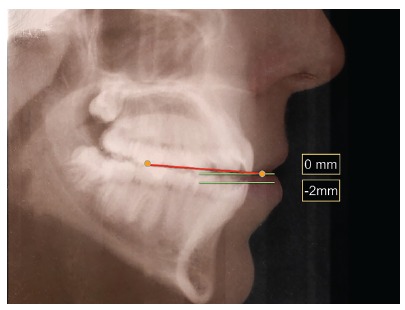
Maxillary incisors touching (0 mm) the FAOP. Ideally, they should be 2 to 4 mm below the FAOP. Mandibular incisors are 2 mm below (-2 mm) the FAOP. Ideally,

Aesthetic clinical examination revealed a low and inverted smile, with a flat incisal line, which resulted in an unpleasant smile and gave the patient an aged appearance (Fig 3). The FAOP lines showed that maxillary incisors touched the plane and mandibular incisors were 2 mm below (Fig 4). However, as previously mentioned, the ideal is that maxillary incisors be 2 to 3 mm below the FAOP, while mandibular incisors touch this plane, in which case incisor exposure would be within the aesthetic standards with lips at rest. This would benefit incisal exposure during speech and probably at smiling, since the smile line would be low.

**Figure 5:**
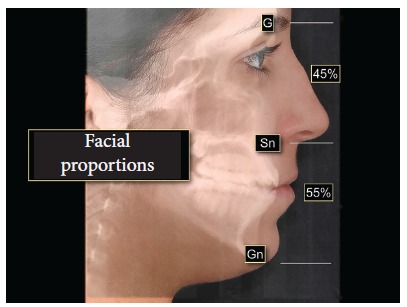
Facial proportions within the normal range 45%/55% (G-Sn/Sn-Gn). they should touch the FAOP.

Moreover the correct interpretation of FAOP involves the evaluation of facial proportions, which, in this case, were within the normal range (limits) of 45/55% (G-Sn/Sn-Gn) between the middle and lower thirds of the face (Fig 5). This observation is important, as the decisions to be taken depend on this evaluation. If facial proportions do not fulfil the standards of normality and aesthetics is jeopardized, then probably orthognathic surgery would be necessary. The idea is that the aesthetic and functional problems are corrected together; however, in cases in which facial proportions are in disharmony, this is not possible. Another possibility to correct open bite would be the intrusion of molars, with a respective counterclockwise rotation of the jaw. In this particular case, this alternative was not an option because the patient would not benefit from molar intrusion, but the problem could be overcome with extrusion of incisors, thus allowing aesthetic and functional benefits.

**Figure 6:**
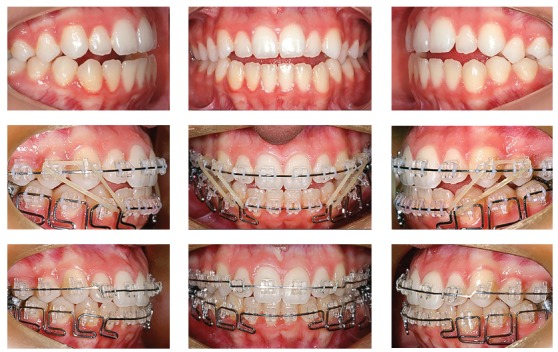
Multiloop Edgewise Arch Wire mechanics (MEAW).

**Figure 7:**
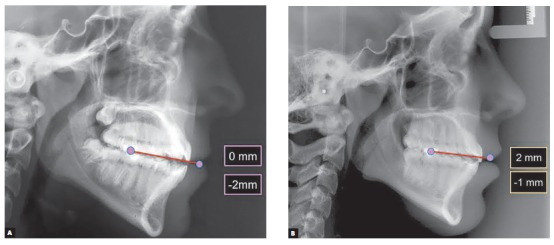
Position of incisors in relation to the FAOP: pre- (A) and post-treatment (B).

**Figure 8:**
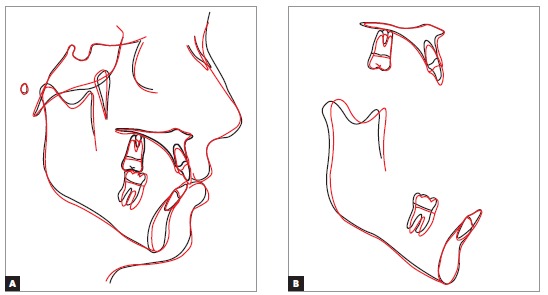
Cephalometric superimpositions of initial (black) and final (red) tracings: total (A) and partial (B).

**Figure 9:**
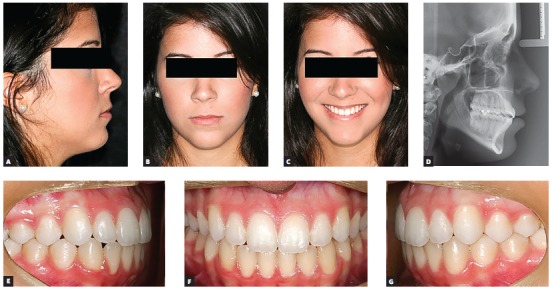
Final photographs: extraoral (A, B, C), radiograph (D) and intraoral (E, F, G).

**Figure 10:**
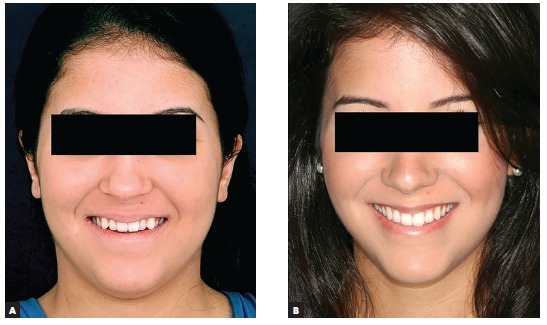
Patient's smile photographs: before (A) and after (B).

**Figure 11:**
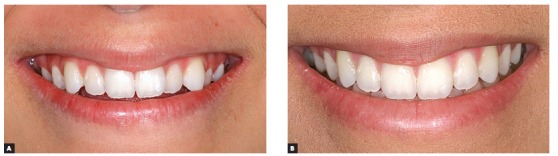
Smile photographs: before (A) and after (B) treatment.

Treatment strategy adopted a mechanical methodology to extrude incisors. The appliance chosen was a multiloop edgewise arch wire (MEAW),^14^ composed of second-order folds in the lower jaw, while in the upper jaw it was made with rectangular 0.019 x 0.025-in TMA wire. Additionally, anterior vertical elastics and Class III elastics (Fig 6) were used. This method was chosen although MEAW requires greater patient cooperation, as it would bring greater control of tooth movement. The extrusive effect of MEAW on mandibular incisors achieved the desired objectives, so that these incisors touched the FAOP. Although mandibular incisors did not fully achieved this goal (Figs 7 and 8), it can be said that they got near enough and did not affect the final results, since overbite was corrected and aesthetics of the smile was improved (Figs 9, 10 and 11).

### Functional Aesthetic Occlusion Plane treatment of deep bite

**Figure 12:**
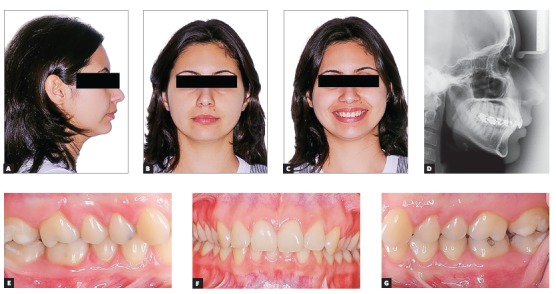
Initial photographs: extraoral (A, B, C), radiograph (D) and intraoral (E, F, G).

The FAOP can also be used to treat patients with deep bite by maintaining maxillary incisors in the most static and aesthetic position possible without compromising orthodontic and functional objectives. In this example, a 23-year-old female patient presented Class II malocclusion. It was more severe on the left side with a vertical overlap of 6 mm between incisors; thus characterizing a deep bite with 2/3 of mandibular incisors exceeding (Fig 12) the planned position of incisors following the FAOP.

**Figure 13:**
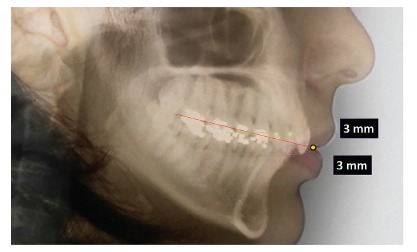
Planning the positioning of incisors. Maxillary incisors are 3 mm below the FAOP in the ideal position, while mandibular incisors are 3 mm above FAOP. Ideally, they should touch the FAOP. In this case, planning involved only the intrusion of mandibular incisors.

Tracing the FAOP on patient's initial radiograph shows that both maxillary and mandibular incisors go beyond the plane by 3 mm each, thus resulting in a 6-mm vertical overlap (Fig 13). In this type of situation, the FAOP provides clues for treatment strategy, since the therapeutic options would be intrusion of incisors, extrusion of molars or surgical correction. In this case, the option was intrusion of incisors, as the patient had a good facial proportion of 50/50. Dismissing the need for surgery and molar extrusion is not recommended for adults due to the possibility of recurrence. Therefore, we opted for incisor intrusion, with greater focus on intrusion of mandibular incisors, which were exceeding the FAOP by 3 mm. The goal, therefore, was that maxillary incisors would bypass the plane by 2 or 3 mm and mandibular incisors would touch the plane. Thus, exposure of incisors would be within the aesthetic standards with lips at rest, which would benefit incisal exposure during speech and probably at smiling.

It was decided to treat Class II on the right side by first premolar extraction, while the left side relationship was treated only with intermaxillary elastics. 

**Figure 15:**
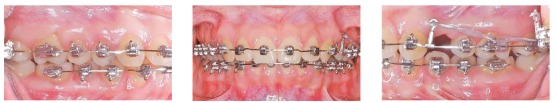
Intraoral photographs of the patient shown in Figures 12 and 13, with 0.016 x 0.022-in S.S. wires inserted. Retraction was performed with sliding mechanics.

**Figure 16:**
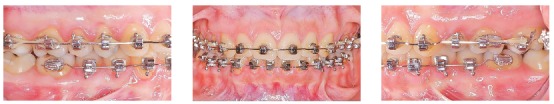
Intraoral photographs of the patient shown in Figures 12, 13 and 14 during the finishing stage of treatment. Note the chain elastics inserted under the finishing wire, as the dimensions of brackets do not allow their insertion over the wire.

Self-ligating 0.018-in slot brackets (MBT prescription, Speed, Strite Industries Limited - Cambridge, Ontario, Canada) were used for treatment. In order to enhance incisor intrusion, mandibular canines were bonded at the same height as mandibular premolars, and mandibular incisors were bonded with a 1-mm height difference of canines (Fig 14). Wire sequence in the upper arch consisted of 0.016-in and 0.016 x 0.022-in superelastic nickel-titanium, in addition to 0.016 x 0.022-in stainless steel wires; while in the lower arch it consisted of 0.014-in and 0.016 x 0.022-in superelastic nickel-titanium, in addition to 0.016 x 0.022-in stainless steel wires. Upper retraction was performed by sliding mechanics, on a 0.016 x 0.022-in stainless steel wire with extensions (Fig 15). Interproximal reduction was carried out on mandibular teeth from canine to canine in order to adjust overjet and Class I relationship. Finishing bends were placed on a 0.016 x 0.022" SS wire in the upper and 0.016-in stainless steel wire in the lower arch (Fig 16).

**showing bracket:**
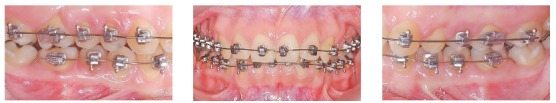
bonding according to plan to correct deep bite only by moving mandibular teeth.

**Figure 17:**
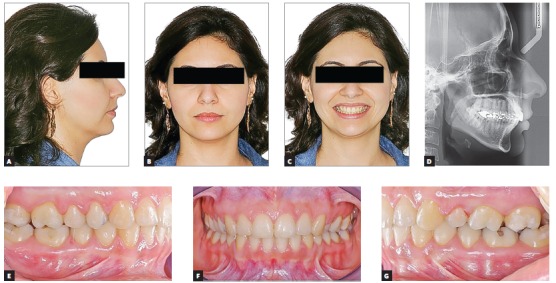
Final photographs: extraoral (A, B, C), radiograph (D) and intraoral (E, F, G).

**Figure 18:**
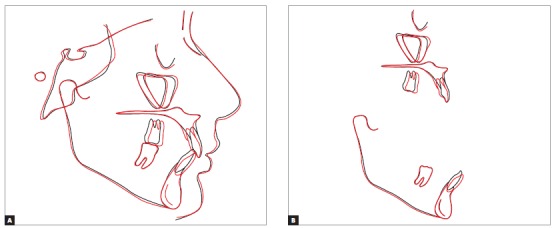
Superimposition of initial (black) and final (red) tracings.

**Figure 19:**
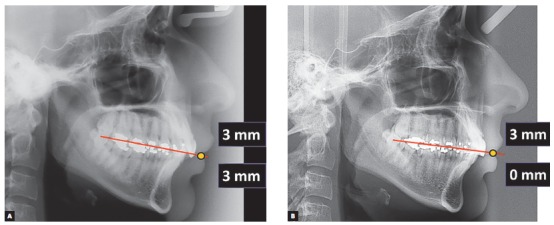
Position of incisors to the FAOP: pre- (A) and post-treatment (B).

**Figure 20:**
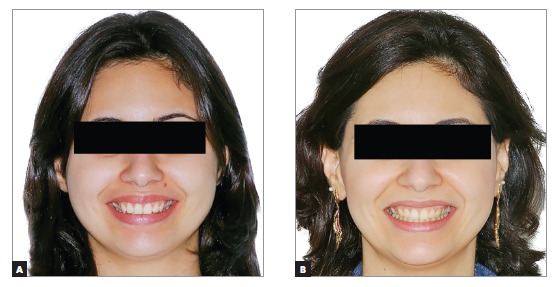
Patient's smile photographs: pre- (A) and post-treatment (B).

**Figure 21:**
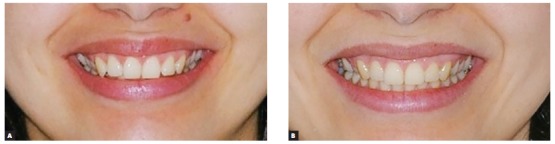
Close-up photographs of patient's smile: pre- (A) and post-treatment (B).

Final photographs reveal good alignment of anterior teeth and a Class I relationship for both canines (Fig 17). A favorable change in the inclination of maxillary incisors was noticeable in the radiographs (Fig 18) due to the prescription with additional torque of maxillary incisors, while a flattening of the lower curve of Spee without much proclination of incisors was noticed due to the negative prescription (-6°) of mandibular incisors added to interproximal wear and retraction. A good positioning of incisors relative to the FAOP was achieved (Figs 19, 20 and 21) as planned, resulting in the correction of deep bite and good aesthetics of patient's smile.

## DISCUSSION

FAOP gives us important information concerning the vertical relationship of incisors with lips at rest and the position of molars in contact, both of which facilitate understanding and limitations of treatment with aesthetic and functional demands (occlusal). It also tells us about the aesthetic possibilities and requirements of the patient, since the relationship of the incisor with the lips indicates if exposure is within acceptable limits according to age, and, at the same time, indicates the reference position of molars and their respective vertical dimension. The idea of ​​a functional aesthetic plane is not new. Burstone and Marcotte^15^ suggested an occlusal aesthetic plane that took into account the position of molars, maxillary incisors and upper lip. The authors stated that maxillary incisors should be 3 mm below the upper lip. Although similar to the plane proposed in the present study, the occlusal plane presented by those authors did not take into account the position of mandibular incisors nor their relationship with the face.

Ideally the incisal edge of mandibular incisors touches the FAOP when the lips are in contact (0 mm). Also, when there is a flattened curve of Spee, the mandibular incisor should have a good vertical overlap with the maxillary incisor due to the need of no contact between them during lateral movements. Additionally, protrusion can promote disocclusion of posterior teeth. This is usually achieved with the incisal edge of mandibular incisors at 0 mm with the FAOP. From a clinical point of view, it would be interesting for the height of mandibular incisors not to exceed the height of the upper lip stomion or the lower lip stomion more than a millimeter (when there is no lip contact).

The use of FAOP facilitates aesthetic and functional diagnosis of cases with rehabilitative and orthodontic requirements, since the use of this plane can easily assess which structures are outside the expected pattern (incisors) and which should be maintained (molars), always taking the patient's age into account. The FAOP must be evaluated in conjunction with the face. Disharmony of the facial thirds might indicate that the aesthetic needs may be beyond the scope of Dentistry with the involvement of bone structures. A ratio between the middle and lower thirds of the face (G-Gn/Sn-Gn) of 50 to 45% to 50-55% is the base parameter^13^ and in which the proportions of 45/55 are the acceptable limits. Facial proportions that are not within these limits may indicate the need for surgery. From an aesthetic point of view, patients with severe facial disharmony do not appear to benefit from orthodontic treatment that only include tooth movement. Although there are reports of using skeletal anchorage to move teeth in vertical direction with consequent morphological changes, the results have not been confirmed in terms of stability, so as to be able to state that this therapy as a safe clinical management practice.^11^ ^,^ ^12^

Many treatment modalities used to correct vertical problems are performed randomly from an aesthetic point of view, as there are no precise parameters to indicate which teeth should be moved; therefore, treatment focuses on the use of biomechanics (treatment plan) instead of aesthetic indicators (diagnosis). Open bites are closed by relying on the extrusion of incisors or intrusion of molars, but this does not take into account the amount of movement required ​​for each group of teeth and the implications of the facial needs and changes. This random strategy results in satisfactory occlusion, closing the open bite, but fails in the aesthetic goals. Exaggerated extrusion of maxillary and mandibular incisors that does not take into account their exposure with respect to the lip and patient's age will not produce agreeable aesthetic results.

As shown, FAOP can serve as a vertical parameter for both open bite and exaggerated overbite. In these two situations, the same parameters and principles are used. An evaluation of the relationship of posterior teeth and the height of molars is made and an evaluation of incisors with the upper lip stomion is also made. In the case of molars, with the exception of patients still in the growth phase, the initial height should be maintained. In relation to incisors, the parameters of maxillary and mandibular incisors with the upper lip stomion are followed: the incisal edge of maxillary incisors should be 2-4 mm below the upper lip stomion and the incisal edge of mandibular incisors should be touching this point.

## CONCLUSION

FAOP can be a complement to an existing and established orthodontic treatment plan, facilitating visualization of functional and aesthetic demands by giving a greater focus to the position of incisors in the relationship among incisors, molars and the upper lip stomion.

Two cases presented in this study demonstrate that FAOP is feasible for clinical use in Orthodontics.
